# Adherence and utilization of short-term antibiotics: Randomized controlled study

**DOI:** 10.1371/journal.pone.0291050

**Published:** 2023-09-05

**Authors:** Basima A. Almomani, Bushra M. Hijazi, Belal A. Al-Husein, Muna Oqal, Lara M. Al-Natour

**Affiliations:** 1 Department of Clinical Pharmacy, Faculty of Pharmacy, Jordan University of Science and Technology, Ar-Ramtha, Jordan; 2 Department of Pharmaceutics and Pharmaceutical Technology, Faculty of Pharmaceutical Sciences, The Hashemite University, Zarqa, Jordan; 3 Department of Pharmacology and Public Health, Faculty of Medicine, The Hashemite University, Zarqa, Jordan; UNITED STATES

## Abstract

Enhancing adherence to medication has the potential to improve clinical outcomes and decrease healthcare cost. The role of clinical pharmacist-led education on adherence to short-term antibiotic has never been investigated in Jordan. This study aimed to evaluate the impact of an educational intervention on antibiotic short-term adherence and to assess the antibiotic utilization pattern. A prospective, single blinded, randomized controlled study was conducted in a tertiary referral hospital in Jordan. Adult patients diagnosed with acute infection and prescribed a short-term antibiotic course (< 30 day) were included in the study. Recruited patients were randomly allocated into control and intervention groups. Pharmaceutical education about the correct use of antibiotic/s was provided to the intervention group. The results showed that penicillins were the most prescribed antibiotics (38.7%) followed by fluoroquinolones (23.9%) and cephalosporines (20.9%). Patients in the intervention group were more likely to be adherent to the prescribed antibiotics compared to control group (OR = 1.445, 95CI% = 1.029–2.030, p = 0.033). Employed patients, less frequent administration of antibiotic, and searching information related to the prescribed antibiotics were factors associated with better adherence to short-term antibiotic (p<0.05). The most common reasons for non-adherence were feeling better and forgetfulness to take medication. These findings highlighted that pharmacist-led educational intervention significantly enhance adherence to prescribed short-term antibiotics which is a major drive to control antibiotic resistance. Initiatives should be adopted to include patient education as a regular element in the medication dispensing process.

**Clinical trial registration**: The trial is registered at ClinicalTrials.gov (identifier: NCT05293977).

## Introduction

In clinical practice, adherence to medications has long been a source of worry, as it has a significant impact on treatment outcomes [1]. Non-adherence to antibiotics or its inappropriate/unnecessary use can lead to the development of antibiotic resistance [2, 3] resulting in negative health consequences and a higher rate of healthcare resources utilization [3, 4]. Various studies have been conducted to measure antibiotic adherence [1–3]. Kandrotaite et al. (2013) measured short-term adherence using both subjective (questionnaire) and objective (pill counts) methods [2]. In a study of ambulatory respiratory infections the different types of antibiotic-taking behavior were examined and self-reported adherence was compared with objectively determined adherence (using electronic monitoring) [1]. Antibiotic adherence has been also assessed for acute infection at the time of discharge from the hospital by phone calling the parents [3].

Patients, treatments, and healthcare practitioners are the major contributors to non-adherence [5]. Patients have a variety of reasons for deviating from the therapeutic plan, either purposeful or accidental [5–7]. For example, patients may (i) decide not fill in their prescriptions, (ii) take more or less medication than advised, (iii) take the medication at the incorrect time, or (iv) stop taking the medication too soon[8]. Inadequate communication between patients and healthcare providers [9] and complex treatment plans may raise the risk of non-adherence as well [10].

Lack of proper information and knowledge about the prescribed antibiotic was revealed to be a significant cause of non-adherence among patients prescribed short-term antibiotic [11]. While information must be communicated to patients in an understandable (or legible) manner, it must also be individualized [5]. In fact, providing patients with specific written or oral information on their antibiotic dosing regimen, appropriate use, possible side effects and importance of adherence has been shown to improve antibiotic adherence [12].

Most studies on medication adherence focused on chronic diseases medications (e.g., antidiabetic, antihypertensive and antihyperlipidemic agents) rather than treatment of acute illnesses, such as infectious diseases requiring short term use of antibiotics [13–19]. The lack of adherence to short term antibiotic has been documented in the literature, however, few studies have focused on educational interventions to improve short-term adherence [12, 20, 21]. In addition, to the best of our knowledge, no studies investigated the impact of the pharmaceutical care intervention programs on short-term antibiotic adherence in the middle eastern region and particularly in Jordan. Consequently, the aim of the current study was to evaluate the impact of pharmaceutical care intervention on antibiotic short-term adherence. A secondary study objective was to assess the utilization pattern of oral antibiotics among outpatient in a tertiary referral hospital in North of Jordan.

## Materials and methods

### Ethics statement

The ethical approval for this research was obtained by the institutional review boards at the Jordan University of Science and Technology (15/126/2019). The trial is registered in ClinicalTrials.gov (identifier: NCT05293977). The consort 2010 checklist is presented in S1 Table and “The authors confirm that all ongoing and related trials for this drug/intervention are registered”. Written informed consent was obtained from all the study participants.

### Study design

A prospective, single blinded, randomized controlled study was conducted at one of the largest tertiary referral hospitals in Jordan, King Abdullah University Hospital (KAUH). The study was carried out during August 2020 till September 2021. The primary outcome determined in this study was the impact of a pharmacist-led educational intervention on adherence to short-term antibiotics among adult patients. A sample of 279 patients in each of the intervention and control groups was required to detect a 15% difference in adherence level between intervention and control group at 95% statistical power and at 5% significant level.

### Patients’ recruitment

All eligible adult patients attending outpatient clinics at KAUH were invited to participate in the study. Adult patients (≥18 years old) diagnosed with an acute infection confirmed by a consultant and prescribed antibiotic pills for short course treatment (< 30 day) at home were included in the study. Patients who were immunocompromised or used prophylactic antibiotics were excluded from the trial. Patients were interviewed by a trained clinical pharmacist (research assistant) in the waiting area of the hospital pharmacy to collect the basic information about the prescribed antibiotics and indications.

### Clinical pharmacist’s intervention

Patients were recruited and randomly allocated into one of the two groups (ratio 1:1); ordinary care (control) group and intervention group. Randomization using a simple technique was adopted; even numbers were assigned for intervention and odd numbers for control. A research assistant was responsible for the enrollment and randomization of patients into the study groups (intervention versus control), in addition to providing the intervention group with pharmaceutical education related to the prescribed antibiotics and following up with both groups by phone calls. To conduct proper education about antibiotics, the 10 most prescribed antibiotics were determined in advance from the hospital records: amoxicillin or amoxicillin/clavulanic acid, ciprofloxacin, levofloxacin, azithromycin, cefuroxime, cephalexin, clindamycin, doxycycline, metronidazole, trimethoprim/sulfamethoxazole. The pharmaceutical education related to each of the aforementioned antibiotics was prepared using the drug information database Lexicomp^®^ [22] and included the followings points (i) mechanism of action and/or use, (ii) correct administration method, (iii) correct timing, (iv) possible adverse effect and self-management intervention methods when faced with side effects, (v) what to do in case of missing any dose. On the other hand, patients in the control group received only a routine pharmaceutical care that was provided by the dispensing pharmacist in the hospital and were interviewed by the research assistant only for data collection. The average time interview was 20–25 minutes for each patient in the intervention group and 10 minutes for the control group.

After obtaining a written informed consent form, a face-to-face interview was conducted to collect the following information at baseline: i) socio-demographic and clinical characteristics, ii) and information related to antibiotic use Two days after completing the antibiotics course regimens, all the patients were followed up by phone to measure adherence by asking them about i) any missing of doses/days of the prescribed antibiotics (subjective method) and ii) number of untaken/remaining pills (objective method). Based on previous studies, participants were considered non-adherent if they failed to follow the number of daily doses and/or duration as prescribed by the physician [2, 23]. In addition, patients were also asked whether they used any other resources of antibiotics information and the reasons of missing doses or not completing the duration of treatment.

### Statistical analysis

Data were analyzed using SPSS software (version 23). Number (percentage) and median (interquartile range, IQR) were calculated for categorical and continuous data respectively. Univariate analysis was conducted using chi square for categorical variables and Mann Whitney test for continuous data. Factors investigated included: study group, age, gender, marital status, education level, employment, monthly income, presence of comorbidity, total number of doses, days antibiotic prescribed, frequency, use the prescribed antibiotic before, and searching information related to antibiotics. Variables with *p* value of less than 0.25 in the univariate analysis were entered in multivariate logistic regression to calculate odds ratio (OR) and 95% confidence interval (95%CI). Intention to treat analysis was adopted for missing data.

## Results

### Demographics

**Fig 1:**
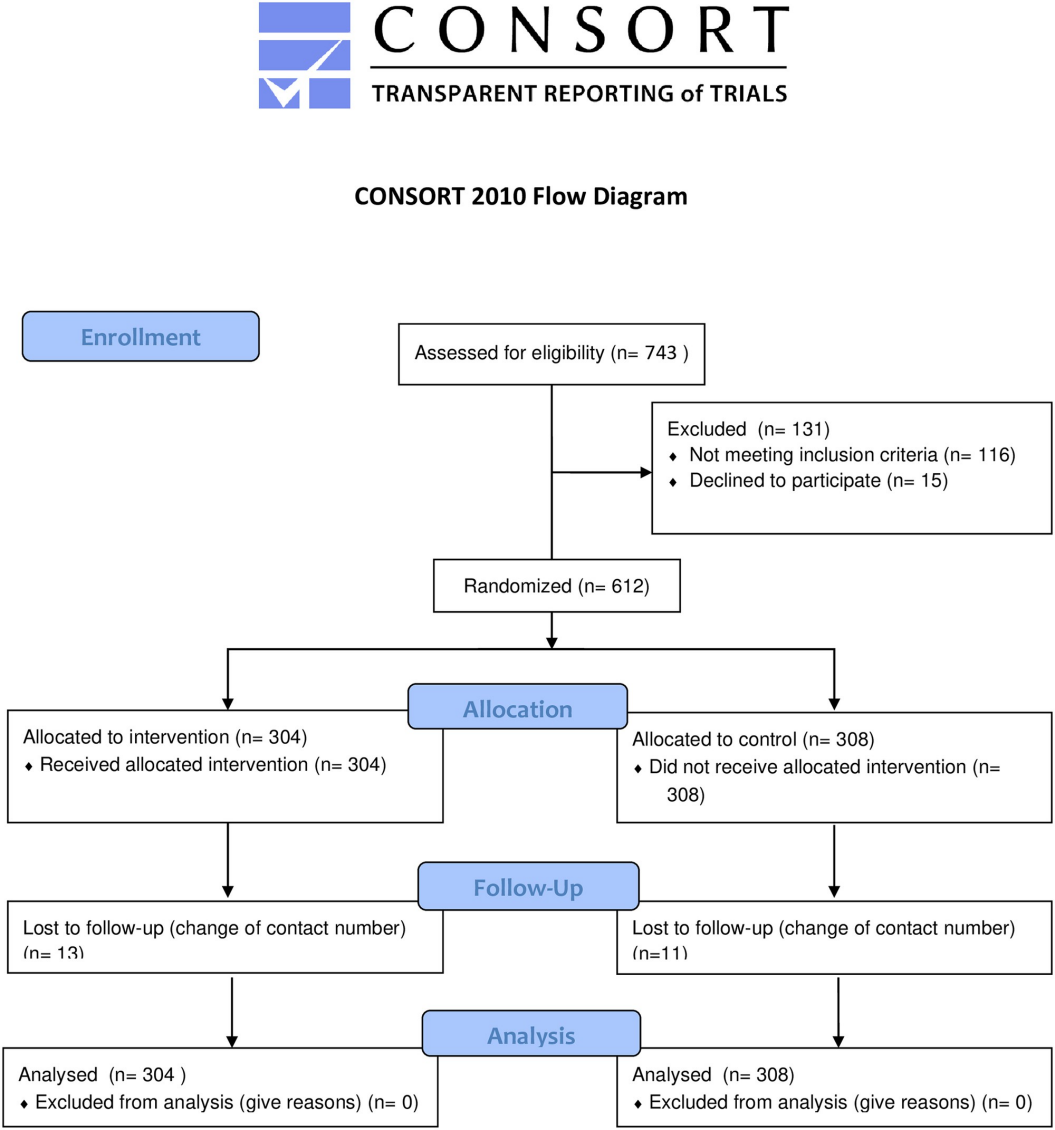
Consort 2010 flowchart.

A total of 743 patients were approached during the study period. Of those, 612 were included in the study. During the follow up, 23 patients [intervention (n = 13) and control (n = 10)] dropped out, 589 patients completed the study [50.4% (n = 297) in the control group versus 49.6%, (n = 292) in the intervention group]. Patients’ recruitment scheme is presented in Fig 1.

The average age of participants was 45 years and about two thirds of them (65.2%) were females. More than three quarters (78.1%) were married, and around half (54.6%) had a university degree. Approximately, a third (36.3%) were employed and 59.6% had a monthly income of ≥500 Jordanian Dinar (JOD; 1 JOD = 1.41 US Dollar). A total of 41.9% of patients had comorbid diseases. Penicillins were the most prescribed antibiotics (38.7%) followed by fluoroquinolones (23.9%) and cephalosporines (20.9%). Approximately, half of antibiotics (48.1%) were prescribed for the duration of 7 days and 62.7% of them with frequency of twice daily. The average number of doses for antibiotic regimen was 20 doses and 64.9% of the patients have used the same prescribed antibiotic previously. Respiratory infection (51.1%) followed by urinary tract infection (UTI, 28.1%) were the most encountered acute infections. There were no significant differences in the baseline demographic and clinical parameters between the intervention and control groups (p value > 0.05). Detailed demographic and clinical data are shown in Table 1.

**Table 1:** Baseline demographic and clinical data of included patient.

Variables^a^	All patients N = 612	Control N = 308 (50.3%)	Intervention N = 304 (49.7%)	P value
Age^b^, years	45 [31–55] Range 18–88	43 [31–55] Range 18–85	45 [32–55.750] Range 18–88	0.467
Gender				0.634
• Female	399 (65.2)	198 (64.3)	201 (66.1)	
• Male	213 (34.8)	110 (35.7)	103 (33.9)	
Marital status				0.486
• Single	134 (21.9)	71 (23.1)	63 (20.7)	
• Married	478 (78.1)	237 (76.9)	241 (79.3)	
Education level				0.526
• School education	278 (45.4)	136 (44.2)	142 (46.7)	
• University education	334 (54.6)	172 (55.8)	162 (53.3)	
Employment status				0.647
• Unemployed	390 (63.7)	199 (64.6)	191 (62.8)	
• Employed	222 (36.3)	109 (35.4)	113 (37.2)	
Monthly income				0.110
• <500 JOD	247 (40.4)	134 (43.5)	113 (37.2)	
• ≥500 JOD	365 (59.6)	174 (56.5)	191 (62.8)	
Presence of comorbidity				0.277
• No	355 (58.1)	185 (60.3)	170 (55.9)	
• Yes	256 (41.9)	122 (39.7)	134 (44.1)	
Indication				0.817
• Respiratory infection	313 (51.1)	158 (51.3)	155 (51)	
• UTI infection	172 (28.1)	85 (27.6)	87 (28.6)	
• Skin infection	54 (8.8)	31 (10.1)	23 (7.6)	
• Fungal infection	26 (4.2)	12 (3.9)	14 (4.6)	
• Others	47 (7.7)	22 (7.1)	25 (8.2)	
Antibiotic categories				0.985
• Penicillins	237 (38.7)	119 (38.6)	118 (38.8)	
• Cephalosporins	128 (20.9)	63 (20.5)	65 (21.4)	
• Fluoroquinolones	146 (23.9)	75 (24.4)	71 (23.4)	
• Azithromycin	55 (9)	29 (9.4)	26 (9.6)	
• Others	46 (7.5)	22 (7.1)	24 (7.9)	
Frequency				0.771
• Once daily	72 (11.8)	38 (12.3)	34 (11.2)	
• Twice daily	384 (62.7)	195 (63.3)	189 (62.2)	
• Three times daily	156 (25.5)	75 (24.4)	81 (26.6)	
Num of doses per regimen^b^	20 [14–28] Range 3–63	20 [14–28] Range 3–42	14 [14–28] 3–63	0.723
Days of antibiotic prescription^b^	7 [7–10] Range 3–21	7[7–10] Range 3–21	7[7–10] 3–21	0.242
Use the prescribed antibiotic before				
• No	214 (35.1)	107 (34.7)	107 (35.4)	0.858
• Yes	396 (64.9)	201 (65.3)	195 (64.6)	

^a^ All data expressed as n (%) of patients unless otherwise indicated.

^b^ Data described as median [Interquartile range]

JOD, Jordanian Dinar; UTI, urinary tract infection

### Utilization of antibiotics

Table 2 shows the usage of antibiotic classes by gender, age groups, presence of comorbidity and indication. Penicillins were the most prescribed antibiotic class among both genders and all age groups except for older adults ≥61 years old. The prevalence of antibiotic use ranged from 41.1% in adults aged 18–30 years to 30.1% in those aged ≥61 years. Among patients with comorbid conditions, penicillins (35.2%) and fluoroquinolones (32.4%) were the most prescribed antibiotics. Penicillins were mainly prescribed for respiratory (55.6%) and skin (51.9%) infections while most fluoroquinolones were prescribed for UTIs (69.2%).

**Table 2:** Antibiotic usage among the participants.

Variables^a^	Antibiotic categories				
	Penicillins	Cephalosporins	Fluoroquinolones	Azithromycin	Others
Gender					
• Female	91 (42.7)	33 (15.5)	70 (32.9)	8 (3.8)	11 (5.2)
• Male	146 (36.6)	95 (23.8)	76 (19)	47 (11.8)	35 (8.8)
Age groups, years					
• 18–30	58 (41.1)	34 (24.1)	15 (10.6)	26 (18.4)	8 (5.7)
• 31–45	73 (41.2)	41 (23.2)	30 (16.9)	9 (5.1)	24 (13.6)
• 46–60	78 (38.8)	39 (19.4)	61 (30.3)	14 (7)	9 (4.5)
• ≥61	28 (30.1)	14 (15.1)	40 (43)	6 (6.5)	5 (5.4)
Presence of comorbidity					
• No	147 (41.1)	78 (22)	63 (17.7)	36 (10.1)	31 (8.7)
• Yes	90 (35.2)	50 (19.5)	83 (32.4)	18 (7)	15 (5.9)
Indication					
• Respiratory infection	174 (55.6)	58 (18.5)	20 (6.4)	53 (16.9)	8 (2.6)
• UTI infection	5 (2.9)	40 (23.3)	119 (69.2)	1 (0.6)	7 (4.1)
• Skin infection	28 (51.9)	16 (29.6)	6 (11.1)	0	4 (7.4)
• Fungal infection	1 (3.8)	8 (30.8)	0	0	17 (65.4)
• Others	29 (61.7)	6 (12.8)	1 (2.1)	1 (2.1)	10 (21.3)

^a^data are presented as n (%).

UTI, urinary tract infection

### Adherence to short-term antibiotics

**Fig 2:**
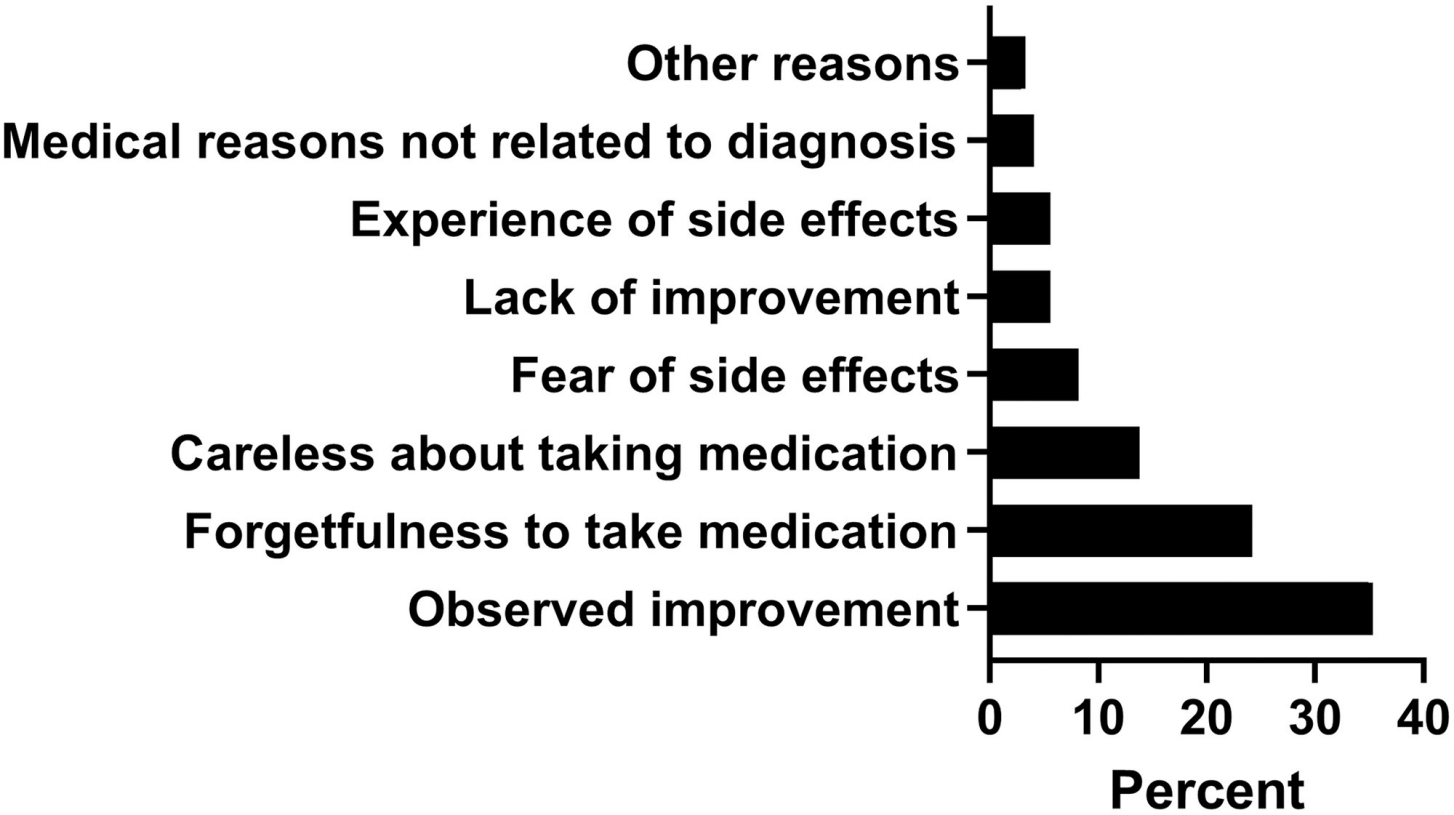
Reasons for non-adherence to short-term antibiotics among the participants.

Out of the 589 patients that completed the follow up phone call, 54% (n = 318) were adherent to the short-term antibiotic. The remaining non-adherent patients reported different reasons for not completing their regimen (duration and/or number of daily doses) such as: felt already better (35.3%), forgot to use the medicine (24.2%) and careless to take their medicine (13.8%). All the reasons stated by the participants are presented in Fig 2. Study group, employment status, frequency of antibiotic dosing, and searching information related to the prescribed antibiotics were found to be significantly associated with antibiotic adherence S2 Table and Table 3. Patients in the intervention group were more likely to be adherent to the prescribed antibiotics compared to control group (OR = 1.445, 95CI% = 1.029–2.030, p value = 0.033). In addition, employed patients were better adherent than unemployed ones (OR = 1.535, 95CI% = 1.074–2.192, p value = 0.019). Furthermore, patients who searched for information related to the prescribed antibiotic were adherent compared to those who did not (OR = 1.664, 95CI% = 1.057–2.622, p value = 0.028). On the other hand, patients who used antibiotic three times daily (OR = 0.094, 95CI% = 0.018–0.497, p value = 0.005) and twice daily (OR = 0.201, 95CI% = 0.081–0.495, p value≤0.01) were less adherent to their prescribed regimens compared to those who used once daily regimen.

**Table 3:** Multivariate analysis of factors associated with short-term antibiotic adherence.

Variable^a^	OR (95CI%)	P value
Study group		
• `Control	Ref	
• Intervention	1.445 (1.029–2.030)	0.033
Marital status		
• Single	Ref	
• Married	0.795 (0.527–1.199)	0.273
Employment status		
• Unemployed	Ref	
• Employed	1.535 (1.074–2.192)	0.019
Frequency		
• Once daily	Ref	
• Twice daily	0.201 (0.081–0.495)	≤0.01
• Three times daily	0.094 (0.018–0.497)	0.005
Num of doses per regimen	1.048 (0.950–1.157)	0.347
Days of antibiotic prescribed	0.887 (0.712–1.104)	0.284
Use the prescribed antibiotic before		
• No	Ref	
• Yes	0.923 (0.640–1.332)	0.668
Searching information related to the prescribed antibiotic		
• No	Ref	
• Yes	1.664 (1.057–2.622)	0.028

^a^ The multivariate analysis was conducted adjusting for variables with p<0.25 in the univariate analysis.

CI, confidence interval; OR, odds ratio

## Discussion

In the current study, the impact of the pharmaceutical care intervention on antibiotic short-term adherence was evaluated and the utilization of oral antibiotics was assessed among outpatients in a tertiary referral hospital in Jordan. This study showed that respiratory infection was the main indication for antibiotics prescription followed by UTI. In a study in Canada, UTI was the main indication for antibiotic prescriptions followed by prescriptions for respiratory infections [24]. This might be attributed to the common practice of using antibiotics in Jordan as they are frequently prescribed for respiratory infections that are viral in nature [25].

Penicillins were the most prescribed antibiotics followed by fluoroquinolones then cephalosporins. A study in Pakistan reported that cephalosporins were the most frequently prescribed class of antibiotics [26]. A study comparing the antimicrobial consumption between a hospital in Northern Ireland and another in Jordan found the prescriptions as follows: penicillins followed by cephalosporins then fluoroquinolones in Ireland, while in Jordan it was cephalosporins followed by fluoroquinolones then penicillins [27]. Another study in Italy reported that penicillins were the most used antibiotic followed by cephalosporins [28]. On this basis, there are variations in the classes of antibiotics most prescribed, however regardless the order, penicillins, cephalosporins and fluoroquinolones were the most prescribed antibiotics. The extensive use of penicillins can also be explained by the high frequency of respiratory infections encountered in the study as penicillins are considered the first line agent for such infections [29]. In addition, penicillins were the most prescribed antibiotics in both genders and in the presence or absence of comorbidities. However, patients who were 61 years old and above received more fluoroquinolones prescriptions than younger ones, while penicillins were the most popular in other age groups. Similar findings were reported by Orlando et al. (2020) reporting penicillins as the most administered antibiotics, and the use of fluoroquinolones increasing by age to become the most prescribed antibiotics in elderly [28].

The study results also showed that pharmaceutical care intervention had a positive impact on antibiotics adherence (OR 1.44), considering other concomitant variables. Previous studies revealed that patients who received educational intervention were more adherent [12, 20]. A meta-analysis of 10 studies involving intervention from pharmacists also showed an improved rate of antibiotic’s adherence with an overall OR of 1.96 [30]. In addition, the present study showed that employed patients tended to be more adherent to their antibiotics. A study in United Kingdom also found employed participants more likely to be compliant with their antibiotic regimen [31]. A possible explanation is that employed patients have limited medical leave, making them more adherent to their prescribed regimen to return to work faster to fulfill their job requirements in a timely manner. Moreover, patients who used antibiotics less frequently were associated with better adherence. Similar finding was observed in previous reports [1, 32]. Furthermore, searching information related to the prescribed antibiotic was identified as a factor of better adherence in the current study. This is supported by a systematic review that reported better knowledge of antibiotics to be associated with higher adherence [33].

Also consistent with previous studies, the most common reasons for non-adherence were feeling better and forgetfulness to take medication [23, 34, 35]. Additionally, being careless about taking the medication was another common reason of non-adherence reported by the study participants. All this can be related to poor knowledge of patients regarding the importance of medication adherence and the consequences of not taking them appropriately. It is clearly highlighted in the literature that when patients have sufficient knowledge about their antibiotics in terms of indication or consequences, they become more adherent [33]. Despite Axelsson et al (2013) reported experiencing side effect as the second common reason for stopping antibiotics [23], this was not a major cause of non-adherence in the current study reported by less than 6% of the participants.

The current study has few limitations. First, 3.8% (n = 23/612) of the participants dropped out from the study period mainly due to changes in the participants’ contact numbers. Second, adherence was self-reported which could be associated with recall bias. To minimize this limitation, adherence was measured two days immediately after the completion of the short-term antibiotic course. Social desirability, another bias associated with the self-reported adherence has been diminished by the presence of intervention and control groups.

## Conclusions

The current study highlighted the patterns of antibiotic utilization among outpatients in a tertiary referral hospital in Jordan. Patients’ education can increase short-term antibiotic adherence. Adherence to antibiotics is different among countries, as various factors contribute to their inappropriate use. Antibiotics misuse and non-adherence remains indeed a major public health issues in Jordan, as there is a non-prescription access to antibiotics. Many initiatives are being adopted to control this problem. Future studies are however needed to measure patients’ behaviors and beliefs that contribute to the inappropriate use of antibiotics as this will assist in proposing different interventions to be examined and implemented.

## Supporting information

S1 Table Consort 2010 checklist. (DOC)

S2 Table Univariate analysis of factors associated with short-term antibiotic adherence. (DOCX)

S1 File Study protocol. (PDF)
