# Supplementary material for: Adherence and utilization of short-term antibiotics: Randomized controlled study
Source: PLoS One. 2023 Sep 5;18(9):e0291050. doi: 10.1371/journal.pone.0291050 (PMC10479900; doi:10.1371/journal.pone.0291050)
Supplement: S2 Table — (DOCX) [file pone.0291050.s002.docx]

**S2 Table. Univariate analysis of factors associated with short-term antibiotic adherence.**

| **Variable^a^** | **Non-adherent**  **N=271** | **Adherent**  **N=318** | **P value** |
| --- | --- | --- | --- |
| Study group   - Control - Intervention | 149 (55)  122 (45) | 148 (46.5)  170 (53.5) | 0.041 |
| Age^b^ | 44 [32-54] | 45 [30-57] | 0.483 |
| Gender   - Female - Male | 176 (64.9)  95 (35.1) | 213 (67)  105 (33) | 0.603 |
| Marital status   - Single - Married | 54 (19.9)  217 (80.1) | 79 (24.8)  239 (75.2) | 0.155 |
| Education level   - School education - University education | 122 (45)  149 (55) | 140 (44)  178 (56) | 0.809 |
| Employment   - Unemployed - Employed | 162 (59.8)  109 (40.2) | 215 (67.6)  103 (32.4) | 0.048 |
| Income   - <500 JOD - ≥500 JOD | 110 (40.6)  161 (59.4) | 124 (39)  194 (61) | 0.693 |
| Presence of comorbidity   - No - Yes | 164 (60.5)  107 (39.5) | 183 (57.7)  134 (42.3) | 0.493 |
| Num of doses per regimen^b^ | 20 [14-28] | 14 [14-21] | 0.001 |
| Days of antibiotic prescribed^b^ | 7 [7-10]  Range 5-21 | 7 [7-10]  Range 3-21 | 0.001 |
| Frequency   - Once daily - Twice daily - Three times daily | 13 (4.8)  176 (64.9)  82 (30.3) | 57 (17.9)  195 (61.3)  66 (20.8) | <0.001 |
| Use the prescribed antibiotic before   - No - Yes | 87 (32.1)  184 (67.9) | 118 (37.3)  198 (62.7) | 0.184 |
| Looking at information related to the prescribed antibiotic   - No - Yes | 232 (85.6)  39 (14.4) | 250 (78.6)  68 (21.4) | 0.028 |

^a^ data are presented as number (percentages).

^b^ median [Interquartile range], range

JOD, Jordanian Dinar
