# Supplementary material for: Adherence and utilization of short-term antibiotics: Randomized controlled study
Source: PLoS One. 2023 Sep 5;18(9):e0291050. doi: 10.1371/journal.pone.0291050 (PMC10479900; doi:10.1371/journal.pone.0291050)
Supplement: S1 File — (PDF) [file pone.0291050.s003.pdf]

TITLE

Short term adherence of antibiotics among Jordanian population

ABSTRACT

Background: Non-adherence to antibiotics may lead to antibiotic resistance which is a significant issue. Additionally, it results in serious health consequences, with a resultant impact on treatment outcomes and consequently more utilization of healthcare recourses. In Jordan, there is a wide access to antibiotics since they can be obtained without prescription from community pharmacies which leads to inappropriate use.

Objective: The current study has two arms. The main goal of the first part study is to evaluate the impact of the pharmaceutical care intervention program antibiotic short-term adherence. The goal of the second part of this study is to assess the practices related to short term adherence of antibiotics in general population in Jordan.

Method: The first study part is an interventional educational part. It includes adults and pediatric patients attending King Abdullah University Hospital (KAUH) and dispensed oral antibiotic for short term treatment (<30 days) from outpatient pharmacy. Patients will be recruited and randomly allocated into one of the two groups; ordinary care group (control) and intervention group. Each patient in the intervention group will be provided with pharmaceutical education about his/her dispensed antibiotic. The second arm of this study is a cross-sectional, questionnaire based, study. All potentially eligible adult and pediatric subjects who recently completed their short course treatment (< 30 day) of oral antibiotic (or more than 50% of course) will be invited to participate in the survey. A face to face interview will be conducted to collect data by using a self-administered questionnaire. The subjects will be approached at different places.

PROJECT GOALS AND OBJECTIVES

The objectives of this study are**:**

- to evaluate the impact of the pharmaceutical care intervention program on the level of adherence to short term antibiotic therapy.
- to assess the prevalence of short-term adherence to antibiotics.
- to explore factors associated with non-adherence to antibiotics.

REVIEW AND ANALYSIS OF RELATED WORK

Medication adherence is defined as the degree to which the behavior of patient taking medication is consistent with agreed recommendations from a healthcare provider (1). The adherence to prescribed regimens has been a concern in clinical practice though it is higher for acute conditions compared to chronic conditions (1). Non-adherence to antibiotics may lead to antibiotic resistance which is a significant issue (2). Additionally, it results in serious health consequences, with a resultant impact on treatment outcomes and consequently more utilization of healthcare recourses (3). Moreover, non-adherence to antibiotics is considered to be one of the factors leading to storing of antibiotics at home which has the potential to lead to self-medication and misuse of antibiotics taking (4). A previous study reported that only 50% of patients were adherent to their antibiotics after returning home (5).

The ability of healthcare professionals to identify the non-adherent subjects to the prescribed/dispensed antibiotics is difficult (1). Previous studies showed that forgetfulness and feeling healthy/better are the common reasons for stopping therapy prematurely (6, 7). Although objective methods (e.g. prescription refill, pill counts) are more reliable to measure the adherence than subjective methods (e.g., patient self-report, questionnaire), they are more expensive and time consuming (2). Subjective methods are simple, practical and useful in clinical practice (1). Different risk factors were reported for non-adherence such as healthcare, condition, therapy and patients related factors (2). Lack of appropriate information and knowledge about prescribed antibiotic, is one of the main causes of antibiotic non-adherence (8). Thus, to enhance short term adherence, patients should be provided appropriate information about antibiotic duration, dosage regimen, and appropriate use. Various studies were conducted to improve the adherence to antibiotics using different educational interventions (9-11).

Despite data regarding short term adherence is available in the literature (4, 5, 7, 8), the use of antibiotics is different among countries as it affected by the availability, accessibility and national polices (12). In Jordan, there is a wide access to antibiotics since they can be obtained without

prescription from community pharmacies which leads to inappropriate use. A recent study reported that about 30% of antibiotics were dispensed without prescription in Jordan (13). Recently, regulations that prohibit antibiotics dispensing in Jordan without prescription had been issued. However, they are not commonly enforced. It is important to assess the Jordanian current adherence behavior toward antibiotics as this will identify the characteristics that affect adherence and the future interventions that are should be addressed. Previous studies in Jordan have focused on assessing the adherence in patients with chronic disease (14-17). There is no information about the adherence to acute conditions.

SIGNIFICANCE OF WORK

Adherence is not only important to guarantee therapeutic effect of antibiotics (or treatment success) but also to decrease antibiotic resistance. Addressing the knowledge, attitudes and practices will help to shape national polices and campaigns targeting the problem of antibiotics resistance.The current study could help to implement preventive actions such as a discharge pharmaceutical consultation for patients with antibiotics. Moreover, accurate estimates of antibiotics adherence will provide better evidence on the consequences, predictors/risk factors, and strategies to improvemedication adherence.

METHODOLOGY

The first arm of the current study is a prospective, single blinded, randomized controlled study interventional educational study. The primary outcome determined in this part is the impact of a pharmacist led educational intervention on adherence to short-term antibiotics among adult patients. A sample of 279 patients per each group will be required to detect 15% difference in adherence level between intervention and control group at 95% statistical power and at 5% significant level. This study includes adults and pediatric patients attending King Abdullah University Hospital (KAUH) and dispensed o ral antibiotic for short term treatment (<30 days) from outpatient pharmacy. Those patients who are immunocompromised, prescribed antibiotic for prophylaxis indication, and used them as part t of H-pylori treatment regimen will be excluded. The patients who fulfill the inclusion criteria will be included in the study only after they have been fully informed and have signed the study consent form. A trained clinical pharmacist will interview the patients in the waiting area of hospital pharmacy to collect the basic information about the prescribed antibiotics and indications. Patients will be recruited and randomly allocated into one of the two groups; ordinary care group and intervention group. Randomization using a simple technique will be adopted; even number will be assigned for intervention and odd number for control. Each patient in the intervention group will be provided with pharmaceutical education about his/her dispensed antibiotic. The aim of the pharmaceutical education intervention is to improve patients' adherence to their prescribed antibiotics. The intervention will include information on the prescribed antibiotics (such as indication, time, duration, possible side effects, missing dose…). Each patient will be interviewed at baseline and then followed up by a phone call, after the completion of antibiotic course. The education will be provided to the 10 most commonly prescribed antibiotics. These antibiotics are determined based on hospital records: A.moxicillin or Amoxicillin/Clavulanic acid, Ciprofloxacin, Levofloxacin, Azithromycin, Cefuroxime, Cephalexin, Clindamycin, Doxycycline, Metronidazole, Trimethoprim/Sulfamethoxazole. The main points of education about antibiotics include (i) mechanism of action and/or use, (ii) correct administration method, (iii) correct timing, (iv) possible adverse effect and self-management intervention methods when faced with side effects, (v) what to do in case of missing any dose. On the other hand, patients in the control group will receive routine

care by the dispensing pharmacist and will be seen by research assistant for data collection only. Two days after completing the antibiotics course regimens, patients in both groups will be followed up by phone to measure adherence by asking them about any missing of doses/days of the prescribed antibiotics (subjective method) and number of untaken/remaining pills (objective method). In the current study, the participants will be labeled as non-adherent if they failed to follow the duration and/ or number of daily doses as prescribed by the physician. These measurements are used based on previous studies (2, 18).

The second part is a cross-sectional, questionnaire based, study. The subjects will be approached at different places (such as outpatients' clinics in hospitals, community pharmacies) from all geographical areas; north, middle and south of Jordan. All potentially eligible adult and pediatric subjects who recently (within the last month) completed their short course treatment (< 30 day) of oral antibiotic (or more than 50% of course) will be invited to participate in the survey. Those patients who are immunocompromised, prescribed antibiotics for prophylaxis indication, and used them as part of H-pylori treatment regimens will be excluded. A face to face interview will be conducted to collect data using the study questionnaire. Informed consent from all participants will be obtained as implied if the participant agrees to fill and returns the questionnaire. This study will adopt a convenience sampling approach. The questionnaire will be pretested for reliability through a pilot study.

In both study arms, the participants' privacy will be preserved throughout the study and the participants' answers will be kept strictly confidential and never associated with any personal details.

Statistical analysis: Following data collection, the responses will be coded and entered into SPSS (version 20). Descriptive statistics will be used to summarize the data for the total sample. The differences in the responses of participants will be examined using a Chi square test (χ^2) test (categorical variables) and t-test or Mann-Whitney *U*-test analysis (continuous variables) as appropriate. Both Kolmogorov-Smirnov Z and Shapiro-Wilk tests will be used to test for normality of continuous variables. Statistical significance will be at $p \leq 0.05$.

LOCATION AND SAFETY CONSIDERATIONS

This study will take place in the faculty of pharmacy/clinical pharmacy department (J.U.S.T), King Abdullah University Hospital (KAUH), respecting the security and safety measures of these institutions. This study does not involve any human biological sample. No potential risks will be associated with the participation in the current study

EXPECTED RESULTS/OUTPUTS

Expected results of the study include the following:

1. Enhance practitioner’s knowledge about the prevalence of short-term adherence among general population.
2. Help decreasing antibiotic resistance, by improving the current practices related to antibiotic prescription
3. Decrease the rate of treatment failure and limit associated consequences and costs.

REFERENCES

1. Osterberg L, Blaschke T. Adherence to medication. N Engl J Med. 2005;353(5):487-97.
2. Kandrotaite K, Smigelskas K, Janusauskiene D, Jievaltas M, Maciulaitis R, Briedis V. Development of a short questionnaire to identify the risk of nonadherence to antibiotic treatment. Curr Med Res Opin. 2013;29(11):1555-63.
3. Sorensen SV, Baker T, Fleurence R, Dixon J, Roberts C, Haider S, et al. Cost and clinical consequence of antibiotic non-adherence in acute exacerbations of chronic bronchitis. Int J Tuberc Lung Dis. 2009;13(8):945-54.
4. Llor C, Hernandez S, Bayona C, Moragas A, Sierra N, Hernandez M, et al. A study of adherence to antibiotic treatment in ambulatory respiratory infections. Int J Infect Dis. 2013;17(3):e168-72.
5. Faure H, Leguelinel-Blache G, Salomon L, Poujol H, Kinowski JM, Sotto A. Assessment of patient adherence to anti-infective treatment after returning home. Med Mal Infect. 2014;44(9):417-22.
6. Vega-Cubillo EM, Andres-Carreira JM, Cirillo-Ibarguen S, Manzanares-Arnaiz C, Moreno-Moreno G, Redondo-Figuero CG. [Non-compliance with the systemic antibiotic treatment prescribed in Primary Health Care emergency departments (Study INCUMAT')]. Semergen. 2017;43(1):4-12.
7. Raupach-Rosin H, Rubsamen N, Schutte G, Raschpichler G, Chaw PS, Mikolajczyk R. Knowledge on Antibiotic Use, Self-Reported Adherence to Antibiotic Intake, and Knowledge on Multi-Drug Resistant Pathogens - Results of a Population-Based Survey in Lower Saxony, Germany. Front Microbiol. 2019;10:776.
8. Chan YH, Fan MM, Fok CM, Lok ZL, Ni M, Sin CF, et al. Antibiotics nonadherence and knowledge in a community with the world's leading prevalence of antibiotics resistance: implications for public health intervention. Am J Infect Control. 2012;40(2):113-7.
9. West LM, Cordina M. Educational intervention to enhance adherence to short-term use of antibiotics. Res Social Adm Pharm. 2019;15(2):193-201.
10. Pham JA, Pierce W, Muhlbaier L. A randomized, controlled study of an educational intervention to improve recall of auxiliary medication labeling and adherence to antibiotics. SAGE

Open Med. 2013;1:2050312113490420.
11. Bilotta C, Lucini A, Nicolini P, Vergani C. An easy intervention to improve short-term adherence to medications in community-dwelling older outpatients. A pilot non-randomised controlled trial. BMC Health Serv Res. 2011;11:158.
12. Zajmi D, Berisha M, Begolli I, Hoxha R, Mehmeti R, Mulliqi-Osmani G, et al. Public knowledge, attitudes and practices regarding antibiotic use in Kosovo. Pharm Pract (Granada). 2017;15(1):827.
13. Haddadin RN, Alsous M, Wazaify M, Tahaineh L. Evaluation of antibiotic dispensing practice in community pharmacies in Jordan: A cross sectional study. PLoS One. 2019;14(4):e0216115.
14. Awwad O, Akour A, Al-Muhaissen S, Morisky D. The influence of patients' knowledge on adherence to their chronic medications: a cross-sectional study in Jordan. Int J Clin Pharm. 2015;37(3):504-10.
15. Sultan K. AlSureehein RMH, Ghaith Abu Alsamen, Khaled M. Alnadi, Wafa S. Alsyoof (2). Poor adherence to inhaler therapy in patients with bronchial asthma: Rates and causes. MIDDLE EAST JOURNAL OF INTERNAL MEDICINE 2015;8(3):4.
16. Jarab AS, Almrayat R, Alqudah S, Thehairat E, Mukattash TL, Khdour M, et al. Predictors of non-adherence to pharmacotherapy in patients with type 2 diabetes. Int J Clin Pharm. 2014;36(4):725-33.
17. Jarab AS, Alqudah SG, Khdour M, Shamssain M, Mukattash TL. Impact of pharmaceutical care on health outcomes in patients with COPD. Int J Clin Pharm. 2012;34(1):53-62.
18. Axelsson M. Report on personality and adherence to antibiotic therapy: a population-based study. BMC Psychol. 2013;1(1):24.
